# The impact of narratives and active video games among black and hispanic children with overweight and obesity: a randomized controlled trial

**DOI:** 10.1186/s12966-025-01756-1

**Published:** 2025-05-26

**Authors:** Amy S. Lu, Tom Baranowski, Tiago V. Barreira, Amy Fleischman, Melanie C. Green, Shirley Y. Huang, I-Min Lee, Lynne L. Levitsky, Farzad Noubary, Debbe Thompson

**Affiliations:** 1 Health Technology Lab, Department of Communication Studies, College of Arts, Media, and Design, Department of Public Health & Health Sciences, Bouvé College of Health Sciences, Northeastern University, 360 Huntington Ave, Boston, MA 02115 USA; 2 Department of Pediatrics, and USDA funded Children’s Nutrition Research Center, Baylor College of Medicine, 1100 Bates Avenue, Houston, TX 77030 USA; 3 Exercise Science Department, Syracuse University, 820 Comstock Ave, Syracuse, NY 13244 USA; 4 Department of Pediatrics, Boston Children’s Hospital, Harvard Medical School, 300 Longwood Avenue, Boston, MA 02115 USA; 5 Department of Communication, University at Buffalo, 359 Baldy Hall, Buffalo, NY 14260 USA; 6 Department of Pediatrics, Tufts Medical Center, Tufts University School of Medicine, 755 Washington Street, Boston, MA 02111 USA; 7 Harvard T.H. Chan School of Public Health, 900 Commonwealth Avenue, Boston, MA 02215 USA; 8 Department of Pediatrics, Pediatric Endocrinology Division, Harvard Medical School, Emeritus Chief, Massachusetts General Hospital, 55 Fruit Street, Boston, MA 02114 USA; 9 Department of Public Health & Health Sciences, Bouvé College of Health Sciences, Northeastern University, 360 Huntington Ave, Boston, MA 02115 USA

**Keywords:** Narrative, Active video games, Physical activity, Body composition, Biomarkers, RCT

## Abstract

**Background:**

Overweight and obesity disproportionately affect Black and Hispanic children who also play more video games. Narratives, coupled with home-based active video games (AVGs), may enhance PA and mitigate these disparities. This study tested the effect of narrative-enhanced home-based AVGs among predominantly Black and Hispanic children with overweight and obesity.

**Methods:**

This 6-month three-group RCT recruited 135 children aged 7–14 from pediatric clinics in Boston, MA (January 2020 - May 2022) during the COVID-19 pandemic. Participants were randomized into: [Narrative + AVG], receiving an Xbox/Kinect with six AVGs interspersed with a narrative animation *Ataraxia* (72 episodes over six months), which accompanied the AVGs; [AVG Only], receiving the Xbox/Kinect and AVGs without narrative animation; and [Waitlist Control], receiving the intervention post-RCT. The primary outcome was objectively assessed daily moderate-to-vigorous PA (MVPA). Secondary outcomes included body composition (fat and lean mass, total region fat), BMI%, fasting insulin, glucose, lipid panel (Cholesterol, HDL, LDL, and Triglycerides), and C-reactive protein. Assessments occurred at baseline, 3, and 6 months. It was hypothesized that [Narrative + AVG] would outperform [AVG Only], which would outperform [Waitlist Control].

**Results:**

79 children completed all three visits (Age = 10.9 *±* 1.7; 63% Boys; 62% Black; 25% Latino; 11% Mixed; and 1% Asian). No statistically significant improvements in MVPA were observed within any condition at 3 or 6 months. A post-hoc exploratory analysis revealed that over the first three months, [Narrative + AVG] increased daily MVPA by 6.8 min compared to [Waitlist Control]. Over the same period, the [AVG Only] group exhibited 815 g less lean mass gain and 7.2 mg/dL lower HDL cholesterol levels relative to the [Waitlist Control].

**Conclusions:**

While neither narrative-enhanced AVGs nor AVGs alone consistently increased daily MVPA across the 6-month RCT, participants in the narrative AVGs group demonstrated greater daily MVPA compared to the control group during the initial three months. During this same period, the AVG-only group exhibited reduced lean mass gain and lower HDL cholesterol levels compared to the waitlist control. The added advantage of narratives was inconclusive, likely due to implementation challenges encountered during the pandemic. These findings highlight the need for addressing these challenges in future research in a fully powered study.

**Trial registration:**

Active Video Games on Physical Activity (Main Trial), NCT04116515. Registered December 25, 2019, https//clinicaltrials.gov/study/NCT04116515.

**Supplementary Information:**

The online version contains supplementary material available at 10.1186/s12966-025-01756-1.

## Background

Sedentary screen media time among children has increased in recent years [1], a trend exacerbated by the COVID-19 pandemic [2]. This increase negatively correlates with physical activity (PA) [3], body composition [4], specifically adiposity (e.g., fat and lean mass, total region fat), BMI percentile [5], and metabolic biomarkers (e.g., triglycerides) [6], all of which are crucial to children’s health and well-being. Even before the pandemic, most PA interventions failed to achieve long-term effects [7, 8]. Key PA challenges include lack of access and motivation [9]. Active video games (AVGs), i.e., games requiring players’ physical movement [10], present an effective, enjoyable, and accessible PA alternative [11]. Currently, all major gaming consoles can be used for AVG [12], but their efficacy can be impaired by children’s declining play motivation [13].

Motivation remains central to PA. Narratives can uniquely motivate AVG play and PA [14]. A narrative refers to two or more events arranged in a temporal order [15]. Integrating PA-motivating narratives with AVGs could enhance engagement and motivation, leading to sustained PA and improved health. Narratives can influence cognition, affect, and behavior through immersion, enabling vicarious personal experiences, and creating deep affection for characters [16]. Despite narrative’s strong potential for behavioral motivation, only 20% of games for health contain narratives, and no AVGs capable of achieving moderate-to-vigorous PA (MVPA) incorporate narratives [17]. Adding narratives to AVGs could foster strong intrinsic PA motivation by recruiting players’ attention, eliciting character identification, and promoting PA perceptions as desirable and fun [18].

Narrative AVGs can be created by enriching existing AVGs with PA-promoting stories that feature characters and plots. Studies have found significant PA improvement among children and young adults using narrative AVGs [19–23]. However, most studies were short-term, ranging from one-time visits to three weeks, with none evaluating long-term impacts over three-to-six months. While narratives can be immersive within a single gaming session, their impact may extend beyond immediate engagement. Ongoing, continuous narratives can enhance adherence to longer-term interventions by creating a “sticky” experience that motivates players to continue AVGs and maintaining PA over time [24]. This sustained engagement is particularly critical for interventions aimed at promoting long-term behavior change. By fostering a deeper connection to the game through evolving storylines, narratives may not only enhance immediate immersion but also encourage consistent participation and PA adherence, ultimately amplifying the intervention’s impact by promoting lasting behavior change. Alternatively, since most AVG consoles can also be used for sedentary activities [25], introducing a new game console into family settings may inadvertently encourage sedentary gameplay, reduce PA, and negatively impact body composition, BMI percentile, and metabolic biomarkers [26]. Such effects can be particularly concerning for racial and ethnic minority youth, who typically spend more time playing video games [27–29]. The critical question emerges: Could integrating narrative with AVGs improve these aforementioned outcomes among racial and ethnic minority children?

The exploration of narrative’s impact on PA, body composition, BMI percentile, and metabolic biomarkers through AVG consoles will allow us to address narrative’s longer-term motivation and explore the potential displacement effect from the gaming consoles. This study is particularly pertinent given the challenges of the recent COVID-19 pandemic, which not only posed significant barriers to PA [2] but also disproportionately impacted Black and Hispanic families [30]. Working with this population to better understand the factors motivating AVG use can help inform the development of future interventions aimed at reducing health disparities.

The primary outcome was objectively assessed daily moderate-to-vigorous PA level. The secondary outcomes included body composition (fat and lean mass, total region fat), BMI percentile, fasting insulin, glucose, lipid panel (Cholesterol, HDL, LDL, and Triglycerides), and C-reactive protein. The exploratory outcomes included additional PA measures (e.g., vigorous PA, moderate PA, brisk walking and daily total steps in brisk walking). Our hypothesis is that the narrative-enhanced AVGs will lead to greater MVPA, improved body composition, BMI percentile, metabolic biomarkers and other PA levels compared to AVGs only, and both will outperform the Waitlist Control, across different phases of a 6-month intervention, including both the first and second halves.

## Methods

### Study design and setting

This study was approved by the Northeastern University’s Institutional Review Board and conducted as a group-randomized controlled trial (RCT) with assessment at baseline, 3, and 6 months. Recruitment occurred between July 2019 and December 2021. The project was not originally designed to be conducted during a pandemic. Nevertheless, the RCT began on January 11, 2020. Due to the pandemic, all research was suspended from March 17, 2020 to September 12, 2020. The final assessment was completed in May 2022. Participants were recruited with parental consent and participant assent and were randomly assigned to one of three groups using permuted-block randomization: [Narrative + AVG], [AVG Only], or [Waitlist Control]. This single-blind trial was conducted on Northeastern University campus and at participant homes in the Greater Boston area. The research project staff members were aware of randomized group conditions; however, participants were not explicitly informed about the nature of the experimental conditions to which they were assigned.

### Recruitment

Participants were recruited from local pediatric clinics: the Optimal Wellness for Life clinic, Boston Children’s Hospital Primary Care Center and Primary Care at Martha Eliot, and Tufts Medical Center, using mass mailings and targeted outreach to overweight or obese patients labeled in the medical record system as they would be at increased risk for metabolic consequences of low PA levels [31]. Parents responded to recruitment materials by completing an online questionnaire with contact and demographic information. A research coordinator followed up for further screening and to confirm eligibility before scheduling the first visit.

Children aged 8–12 years with a BMI-for-age percentile ≥ 85% were included. Due to the pandemic shutdown, the research team expanded the age range from 8 to 12 to 7–14 to accommodate those affected by the pandemic-related research suspension​ [32]. Pilot work identified narrative and the AVG games to appeal to this broader age range as well. Exclusion criteria included non-English speakers, prior experience with selected AVGs, vision/hearing impairments, intellectual disabilities affecting assent or task completion, and medical or physical issues preventing AVG play.

### Intervention

The RCT began with a pilot test, during which the protocol was slightly modified, such as sending multiple communication messages for scheduling and creating an additional instruction sheet for Xbox setup, to improve intervention adherence [33]. Subsequently, additional modifications were made to address unanticipated challenges posed by the COVID-19 pandemic [32, 34]. Despite these adjustments, the majority of the intervention components remained unchanged. Details of the protocol’s evolution are available on ClinicalTrials.gov [35].

During the first visit, participants in both the [Narrative + AVG] and [AVG Only] groups received an Xbox/Kinect bundle preloaded with *Shape Up* (Ubisoft Montreal, Canada), the first of six AVGs included in the RCT. Setup instructions and guidance for downloading five additional AVGs over the six-month period were provided to maintain variety and prevent boredom. The [Narrative + AVG] and [AVG Only] groups only were instructed to play AVGs for 30–60 min daily, with a fridge magnet provided as a visual reminder of the time requirements. Families signed an agreement to ensure at least 60 min of weekly AVG play during the RCT, and parents were encouraged to support adherence. The [Waitlist Control] group also signed an agreement to complete at least 60 min of PA weekly.

Although children in both AVG groups could play with others, they were advised to focus on their own playtime, as only their activity levels were tracked. Over six months, additional AVGs [*Zumba Fitness: World Party* (Zoë Mode, Brighton, England), *Beatsplosion* (Virtual Air Guitar Company, Helsinki, Finland), *Fruit Ninja* (Halfbrick Studios, Brisbane, Australia), *Kinect Sports Rivals* (Rare, Twycross, England), and *Kung Fu for Kinect* (Virtual Air Guitar Company, Helsinki, Finland)] were introduced after *Shape Up*, allowing children greater choice in game selection, all of which were pretested to elicit MVPA under normal play conditions.

Study staff regularly checked in with participants to monitor progress, troubleshoot devices, and answer questions. Participants who failed to meet the playtime requirement were contacted by study staff. Reminder contacts (email, phone, or text) for the [Waitlist Control] participants were matched to the weekly average sent to the [Narrative + AVG] and [AVG Only] groups.

The research team originally developed an algorithm to monitor AVG gameplay and track adherence using Xbox.com play logs. Objective play data were accessible through participants’ accounts, and an automated script was created to download and analyze this data. However, changes made by AVG developers to game code within the Xbox.com library rendered tracking objective play data impossible two months into the RCT. Despite multiple attempts to contact developers and Microsoft, the issue remained unresolved during the pandemic, preventing effective documentation of gameplay and usage data.

For AVG selection, children started with one game in the first month, expanding to six games by Month 6. While they were encouraged to play to the best of their ability, the aforementioned technical limitations prevented real-time documentation of gameplay intensity or engagement. Nonetheless, the increasing variety of AVG options provided children with greater flexibility in choosing games, provided they successfully downloaded and installed them.

The key distinction between the [Narrative + AVG] and the [AVG Only] groups was that the former also received instructions to download the narrative animation series, *Ataraxia* (72 episodes across six seasons), onto the Microsoft OneDrive app preinstalled on their Xbox consoles. The *Ataraxia* animation series was co-designed with a separate group of diverse children to ensure inclusive narrative design, identifying preferred narrative genre (a Sci-Fi story with virtuous characters, extraordinary actions, interesting plots, super powers, and engaging cliffhangers) [36], racial presentations (characters who are racially ambiguous with fantastical hair and eye color schemes) [37] and body shapes (characters who are also overweight or obese) [38]. Additionally, we have found the optimal plot structure to be serial instead of episodic [22].

**Fig. 1:**
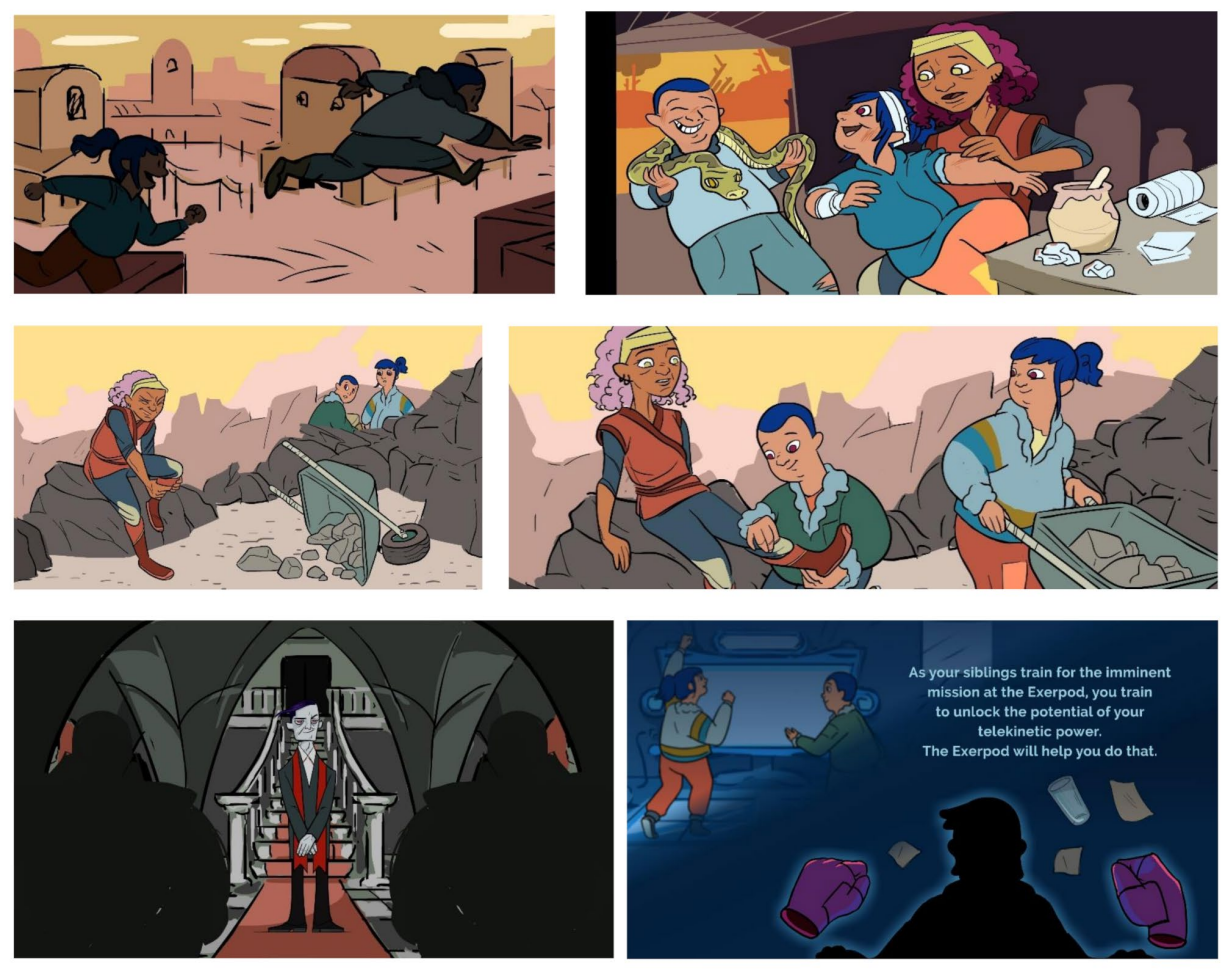
Screenshots of the *Ataraxia* animation

The *Ataraxia* narrative is set in a dystopian future where a woman adopts twin siblings with superpowers and raises them alongside her own child. An evil ruler kidnaps the twins to build an army for his kingdom. The woman’s child, addressed as “you” and depicted as an androgynous shadow for children’s self-projection, grows up inspired by the twins and begins to develop superpowers through AVG exercise. The goal is to control these abilities to rescue the twins and save the world (see Fig. 1 for screenshots of the main characters).

Child players are encouraged to identify with the “you” character and play the AVGs. At the end of each episode, a “call to play” panel encourages the children to engage in the AVGs. Additionally, at the end of each season (six total), the “you” character acquires a new superpower, which enhances the character’s growth and abilities throughout the story, motivating continuous AVG participation and engagement.

The [Narrative + AVG group] could watch the animation episodes between AVG gameplay (the first three episodes of season one were preloaded). The subsequent animation episodes had to be downloaded at a later time, approximately three episodes per week. The [Waitlist Control] group did not have access to a project Xbox, any AVGs, or narrative until after they completed the final data collection.

The narrative structure, with its incremental acquisition of superpowers and immersive storytelling, aimed to motivate children to sustain their PA levels by making the process engaging and rewarding. The calls to play panels at the end of each episode served as direct prompts to play the AVGs, reinforcing the connection between the narrative and PA.

### Outcome assessments: primary, secondary, and exploratory measures

Children’s PA behavior was assessed using an ActiGraph GT3X + Triaxial accelerometer (ActiGraph, Pensacola, FL) attached to their nondominant hip. After completing each of the visits, they were provided with accelerometers initialized for seven days starting at midnight (00:00 am) of the day of the visit, thus starting around 12 h post visit. Participants were instructed to wear the device for the full week on their nondominant side, except during water activities, and then mail the accelerometers to the lab in a prepaid package. The device has been validated and is a reliable assessment tool for children’s PA measurement with good compliance [39]. The accelerometer recorded for seven days at a frequency of 80 Hz starting at midnight of the visit day, including a weekend day [40]. PA data were considered valid if participants had at least three days with ten hours or more of wake-wear per day [41]. ActiLife software was used to process the PA data using established protocols in 60- and 15-sec epochs with the low-frequency filter extension [42]. Nocturnal sleep duration was estimated using a published automated algorithm [43, 44], and the time between in and out of bed were removed from the analysis. Wake nonwear time was identified as 20 consecutive minutes of zero counts using the 60-sec epoch [45]. Awake time was analyzed using the 15-sec epoch. Using the Evenson cut points [46], time spent in daily MVPA was calculated. Since MVPA includes both moderate PA (MPA) and vigorous PA (VPA), which have different influence on children’s development [47–49], as additional exploratory measures, we also examined MPA and VPA separately, as well as brisk walking, which may uniquely benefit children’s aerobic fitness [50–52]. Since most AVGs involve continuous and rhythmic movements that resemble the patterns of brisk walking, a cadence threshold of ≥ 100 steps/min was used to determine the time and number of steps spent at brisk walking [53].

Secondary outcomes included: body composition, assessed using Dual-energy X-ray absorptiometry (DEXA) by a certified DEXA technician (G.E. Lunar Corp. Madison, WI). Participants lay supine with arms at their sides, palms down, and thighs separated. Scans were analyzed using the whole-body fan beam method to assess total region fat (%). BMI% was calculated based on the height and weight information collected during the scan.

Participants fasted for 8–12 h before each of the three visits, and consumption was documented. Blood was drawn via venipuncture, with one EDTA tube for plasma and one serum tube (8.5–10 mL each) collected. EDTA tubes were centrifuged at 4 °C at 2300 RCF for 15 min, while serum was clotted for 30 min at room temperature before centrifugation at 3000 RCF for 15 min. Samples were aliquoted and stored at − 80 °C. Insulin and C-reactive protein were measured by Enzyme-Linked Immunosorbent Assay (ELISA), while glucose and lipid panels (Cholesterol, HDL, LDL, and Triglycerides) were analyzed using standard biochemical assays.

Our primary and secondary outcomes were specified in the RCT Registration (NCT04116515), with primary assessment timepoints designated at both 3 and 6-months. This approach aimed to evaluate outcomes comprehensively, capturing effects both mid-way through and at the conclusion of the intervention. Additionally, examining the two segments of the RCT allowed us to capture how children’s experiences evolved over time, providing valuable insights into engaging with underrepresented youth. Nevertheless, because the comparison between Month 3 and Month 6 was not explicitly defined a priori in our protocol, we present this analysis to the Supplementary Analysis section. These exploratory comparisons were not pre-specified in the registration.

### Intervention engagement and acceptance

A game experience questionnaire [54] was administered to participants in Conditions [Narrative + AVG] and [AVG Only]. Participants in the [Narrative + AVG] condition also rated narrative immersion [16], story enjoyment (e.g., “I liked this story”) and interest in the narrative’s development (e.g., “I would like to keep watching more episodes of this story”) on a 5-point scale, with 1 indicating “Not at All or Disagree” and 5 indicating “Extremely or Agree.” To confirm engagement, participants were periodically asked narrative-specific questions that required viewing the animation (e.g., “What color is [A Supporting Character’s Name]’s hair? Purple/Orange/Green/Blue”). Additionally, project coordinators gathered informal feedback during interactions with families in Conditions [Narrative + AVG] and [AVG Only].

### Sample size

**Fig. 2:**
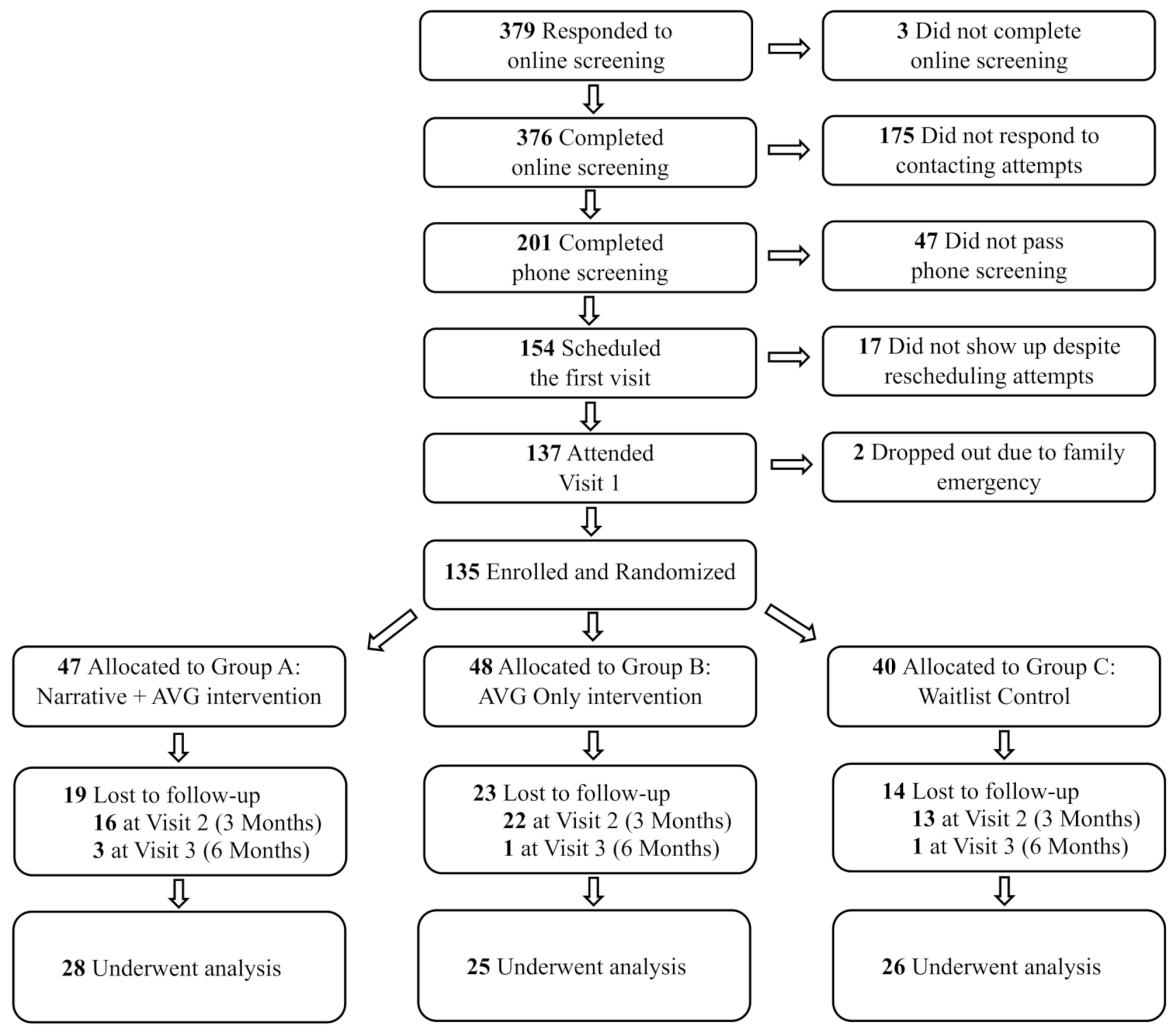
Recruitment and flow of participants

The initial planned sample size was 210 (70 per condition) with an alpha of 0.05, aiming for more than 96% power to detect a mean daily MVPA change of 5 min per day and a 0.5% change in Total Region % Fat, assuming standard deviations of 8 min per day and 0.3%, respectively, and an 80% retention rate. Despite the research team’s efforts to preserve funding and expedite the project after the research resumed, the pandemic and associated interruptions led to severe challenges to recruitment and retention. Of the 376 parents who initially enrolled, 135 children were enrolled and randomized at visit one; 84 attended the second visit, and 79 completed all three visits (Fig. 2) before the funding was exhausted, terminating data collection and decreasing the study’s power to 60%.

### Statistical methods

Due to the significant dropout rate between the first and second visits caused by the pandemic suspension, and a much smaller decrease between the second and third visits, the analysis was focused on the 79 participants who completed all three visits. Linear mixed effects models, incorporating fixed effects for time, group, and time*group interaction, along with subject-specific random intercepts and slopes, were fitted to test the main hypothesis across different RCT stages. Visit-specific mean value imputation [55] was conducted for all primary, secondary, and exploratory variables to preserve statistical power, given the low missing value rate (1–5%) [56–58]. While focusing on participants who completed all three visits, applying mean imputations, and comparing the effects both mid-way through and at the conclusion of the intervention represented departures from the original analytical plan, these adjustments for the post-hoc exploratory analysis were deemed reasonable and necessary under the unprecedented circumstances described. Additionally, a sensitivity analysis was conducted to control for baseline PA levels (Physical Activity Questionnaire for Older Children, PAQ-C) [59] and Children’s Social Desirability [60], which measures demand characteristics to mitigate reporting bias. Social desirability bias occurs when participants provide responses they perceive as socially acceptable rather than truthful. This bias is particularly relevant in studies involving behaviors like PA, where participants may overreport adherence or underreport less favorable behaviors. Controlling for this bias is crucial in reducing potential inaccuracies. Finally, corrections for multiple comparisons were performed using the Benjamini–Hochberg procedure [61]. All analyses were conducted using SAS PROC Freq/Means/Mixed (SAS 9.4, Cary, NC). Data are expressed as mean [Standard Error (SE)] unless otherwise indicated.

## Results

### Participants

The demographic and clinical characteristics of the 79 participants at baseline, categorized by treatment condition, are listed in Table 1. No significant between-group differences were observed among conditions, between the 79 who completed all three visits and the 135 who completed at least one visit, or between the originally planned 8–12 age group and the expanded 7–14 age group due to the pandemic shutdown (*p*s > 0.54). From the 135 enrolled participants, 19 dropped out from group A (40.43%), 23 from group B (47.92%), and 14 from group C (35%). The dropout rates were analyzed using both the Chi-square test and Fisher’s exact test, with no statistically significant differences observed among the three groups (*p*s > 0.45). Additionally, no baseline differences were identified among the groups (*p*s > 0.10).

**Table 1:** Demographic and clinical characteristics of participants at baseline by condition, values expressed as mean ± sd

	Total (*N* = 79)	Narrative + AVG (*n* = 28)	AVG Only (*n* = 25)	Waitlist Control (*n* = 26)	*p*
Age (years)	10.93 ± 1.68	11.22 ± 1.61	10.94 ± 1.73	10.59 ± 1.70	0.39
Boys/Girls	50/29	20/8	14/11	16/10	0.50
Height (cm)	152.72 ± 11.77	154.08 ± 12.23	151.61 ± 11.65	152.32 ± 11.71	0.74
Weight (kg)	63.45 ± 16.56	64.79 ± 17.98	63.50 ± 17.04	61.91 ± 14.87	0.82
Body Mass Index (BMI) percentile	96.38 ± 3.48	96.42 ± 2.67	96.21 ± 3.89	96.49 ± 3.96	0.96
Social desirability	3.33 ± 0.87	3.29 ± 0.94	3.32 ± 0.80	3.38 ± 0.90	0.92
Race, n* (%)					0.09
Asian	1 (1.3)	0 (0)	0 (0)	1 (3.8)	
Black	49 (62)	16 (57.1)	18 (72.0)	15 (57.7)	
Latino	20 (25.3)	6 (21.4)	4 (16)	10 (38.5)	
Mixed	9 (11.4)	6 (21.4)	3 (12)	0 (0)	
Native American	0 (0)	0 (0)	0 (0)	0 (0)	
White	0 (0)	0 (0)	0 (0)	0 (0)	
Other	0 (0)	0 (0)	0 (0)	0 (0)	
Household Highest Education Level, n* (%)					0.83
6th Grade or less	1 (1.3)	0 (0)	1 (4.0)	0 (0)	
8th Grade or less	2 (2.5)	1 (3.6)	1 (4.0)	0 (0)	
Some High School	3 (3.8)	1 (3.6)	0 (0)	2 (7.7)	
High School	7 (8.9)	3 (10.7)	2 (8.0)	2 (7.7)	
Technical School	7 (8.9)	3 (10.7)	2 (8.0)	2 (7.7)	
Some College	24 (30.4)	11 (39.3)	5 (20)	8 (30.8)	
College Graduate	26 (32.9)	7 (25%)	10 (40)	9 (34.6)	
Post Graduate Study	9 (11.4)	2 (7.1%)	4 (16)	3 (11.5)	
Household Income, n* (%)					0.20
Less than $20,000	16 (20.3)	4 (14.3)	5 (20)	7 (26.9)	
$20,000–39,999	18 (22.8)	9 (32.1)	5 (20)	4 (15.4)	
$40,000–59,999	17 (21.5)	9 (32.1)	2 (8)	6 (23.1)	
$60,000–79,999	10 (12.7)	3 (10.7)	4 (16)	3 (11.5)	
$80,000-100,000	9 (11.4)	0 (0)	6 (24)	3 (11.5)	
More than $100,000	9 (11.4)	3 (10.7)	3 (12)	3 (11.5)	
Type of Residence, n* (%)					
Single Family House	19 (24.1)	5 (17.9)	8 (32)	6 (23.1)	0.53
Townhouse	11 (13.9)	2 (7.1)	3 (12)	6 (23.1)	
Condominium	2 (2.5)	1 (3.6)	1 (4)	0 (0)	
Apartment	44 (55.7)	18 (64.3)	13 (52)	13 (50)	
Other	3 (3.8)	2 (7.1)	0 (0)	1 (3.8)	

*: 100% for each condition

SD: Standard Deviation

### Outcome variables

**Fig. 3:**
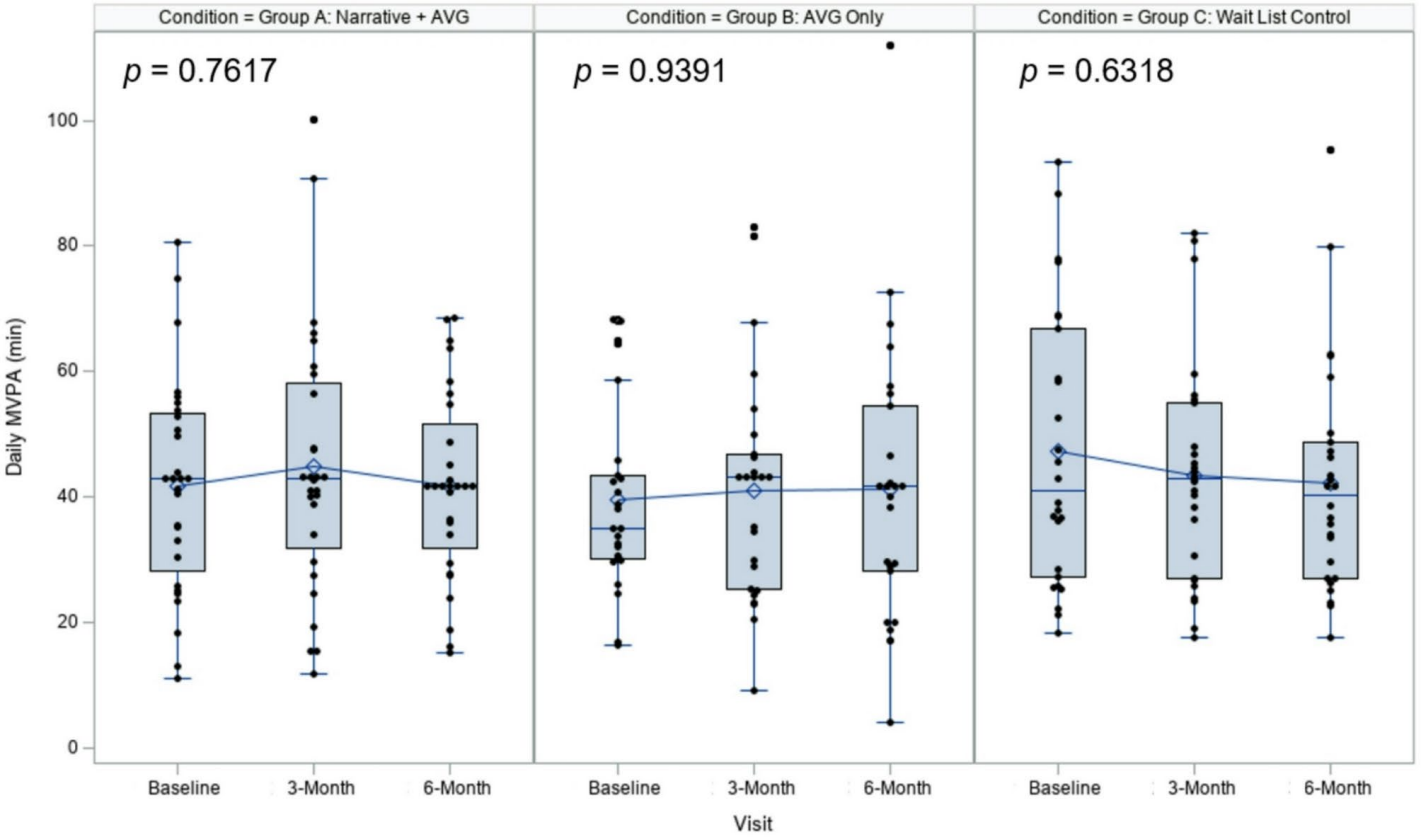
Box plots of the primary outcome across conditions

Results for the primary, secondary, and exploratory outcomes are reported in Table 2. We did not observe a statistically significant difference in the primary outcomes at both 3 months or 6 months within any of the three conditions (*p*s > 0.63). Figure 3 presents the box plots of the primary outcome across the three conditions. Additionally, the baseline MVPA levels were relatively high compared to national prevalence estimates [62] across all three groups, with no significant differences observed between the groups (*p* > 0.3), suggesting that the sample may have been more active than the general population from the outset. However, our post-hoc exploratory analysis indicated that over the first three months, the [Narrative + AVG] group had significantly more daily MVPA (net difference, 6.8 [4.9; 95% CI, 1.3–14.8], minute/day; *p* = 0.02) than [Waitlist Control] but not [AVG Only].

**Table 2:** Between-Condition differences in physical activity levels, body composition, BMI percentile, and metabolic biomarkers through linear mixed model

	Group A: Narrative + AVG			Group B: AVG Only			Group C: Wait List Control			Btw-Cond. Diff: A vs. B B-3 M Change	Btw-Cond. Diff: A vs. B B-6 M Change	Btw-Cond. Diff: A vs. C B-3 M Change	Btw-Cond. Diff: A vs. C B-6 M Change	Btw-Cond. Diff: B vs. C B-3 M Change	Btw-Cond. Diff: B vs. C B-6 M Change
Study Outcome	B	3 M	6 M	B	3 M	6 M	B	3 M	6 M						
**Primary Outcome**															
Daily MVPA (min)	41.8 (17.3)	44.9 (20.9)	41.8 (14.9)	39.6 (14.9)	41.1 (18.3)	41.3 (22.8)	47.3 (22)	43.5 (17.8)	42.3 (18.2)	1.5 (4.9)	-1.7 (4.2)	6.8* **(4.9)**	5 (4.2)	5.3 (5.0)	6.7 (4.3)
**Secondary Outcomes**															
Total Fat (g)	26269.9 (11513.8)	26816.5 (11584.1)	27094.5 (11032.1)	27101.5 (10689)	26745.1 (11173.7)	26746.6 (11855.4)	25503.5 (8479.7)	26338.2 (8968.2)	27786.4 (9755.6)	903.0 (943.2)	1179.5 (1258.4)	-288.1 (933.6)	-1458.2 (1245.6)	-1191.1 (960.2)	-2637.7 (1281.0)
Total Lean (g)	35,416 (8876.3)	37055.5 (9310.5)	37922.7 (10079.9)	34269.6 (6809.4)	35463.2 (7744.8)	36300.7 (8028.3)	33848.6 (7418.5)	35857.3 (9376.8)	36813.2 (10348.6)	445.9 (974.7)	475.6 (1260.0)	-369.2 (964.7)	-457.9 (1247.1)	-815.1* (992.2)	-933.5 (1282.6)
Total Region Fat (%)	40 (8.5)	39.6 (8.9)	39.7 (8.8)	41.5 (7.5)	40.5 (7.9)	39.8 (9)	40.9 (6.2)	40.5 (6.2)	41.2 (6.2)	0.6 (0.7)	1.4 (0.9)	-0.1 (0.7)	-0.5 (0.6)	-0.7 (0.8)	-1.9 (1.0)
BMI %	96.4 (2.7)	95.9 (3.7)	96.1 (3)	96.2 (3.9)	96.3 (3.2)	95.8 (3.9)	96.5 (4)	96.9 (3.1)	96 (4.9)	-0.6 (0.6)	0.1 (0.5)	-1 (0.5)	0.2 (0.6)	-0.4 (0.5)	0.1 (0.7)
Fasting Insulin	16.6 (11.5)	16.9 (10)	16.8 (16.4)	13.1 (7.1)	21 (17.2)	14.3 (8.5)	18.6 (12)	23.8 (27.6)	20.9 (22.5)	-7.7 (4.6)	-1.1 (3.2)	-5 (5.2)	-2.2 (4.5)	2.7 (6)	-1 (4.4)
Glucose	91.3 (9.3)	89.9 (8.8)	88.8 (11.1)	89.5 (8.7)	91.9 (6.8)	89.7 (8.1)	93.8 (23.3)	96.8 (32)	96.7 (42.9)	-3.8 (2.6)	-2.6 (2.9)	-4.4 (3)	-5.3 (4.9)	-0.6 (2.9)	-2.7 (5.1)
Cholesterol	164.3 (23.7)	159.5 (29.9)	158.2 (21.1)	163.1 (33.2)	153.7 (28.4)	160 (39.4)	163.2 (32.3)	164.7 (35.4)	159.6 (36.5)	4.6 (6.1)	-3 (5.3)	-6.2 (5.4)	-2.4 (5.6)	-10.8 (6.9)	0.6 (6.5)
HDL cholesterol	51.2 (10.4)	52.1 (14.4)	52.9 (10.3)	49.3 (13.7)	47.3 (9.8)	48 (9.9)	48.8 (10.3)	54 (12.2)	51.8 (13.6)	2.9 (2.8)	3 (3)	-4.3 (2.4)	-1.3 (2.5)	-7.2* **(2.5)**	-4.3 (3.1)
LDL cholesterol	95.7 (22.8)	89 (26.2)	89.1 (20.5)	96.7 (25.1)	89.7 (26.1)	94.3 (32.2)	96 (26.2)	92.2 (27.1)	89.2 (27.7)	0.4 (5.2)	-4.2 (4.3)	-2.8 (3.7)	0.3 (4.5)	-3.2 (5.7)	4.5 (5.1)
Triglycerides	84.9 (32.2)	97.2 (64.4)	77.3 (34.2)	80.1 (23.7)	79.8 (23.5)	86.1 (28.3)	92 (34.2)	92.5 (44.9)	93.1 (37.3)	12.6 (13)	-13.6 (8.7)	11.8 (12.8)	-8.7 (9.6)	-0.8 (9.4)	4.8 (10)
C-reactive protein	3.2 (4.2)	3.7 (3.5)	4 (4.7)	3.3 (2.4)	3.6 (3)	2.6 (2.2)	2.8 (2.9)	2.7 (2)	3 (3.6)	0.2 (0.6)	1.6 (0.9)	0.5 (0.5)	0.7 (0.9)	0.3 (0.6)	-0.9 (0.6)
**Exploratory Outcomes**															
Daily Vigorous PA (min)	10.1 (5)	12.7 (10.7)	11.3 (7.8)	10.7 (5.8)	9 (5)	9.9 (7.4)	12.9 (9.2)	11.5 (6.8)	10.7 (6.8)	4.3 (2.1)	2 (1.9)	4 (2.1)	3.4 (1.8)	-0.3 (2.1)	1.5 (1.9)
Daily Moderate PA (min)	31.7 (13.3)	32.2 (15.3)	30.5 (11.5)	28.9 (10.5)	32.1 (13.7)	31.4 (15.7)	34.3 (13.9)	32 (12.8)	31.6 (13.2)	-2.8 (3.8)	-3.7 (3.0)	2.8* **(3.8)**	1.5 (3.0)	5.6* **(3.9)**	5.2 (3.1)
Daily Brisk Walking (min)	5.3 (6.2)	6.9 (10.8)	5.3 (8.5)	4.4 (3.8)	4.1 (3)	4.2 (4.6)	4.1 (4.2)	3.1 (3.4)	3.1 (2.6)	1.8 (1.9)	0.1 (1.9)	2.5 (1.8)	0.9 (1.8)	0.6 (1.9)	0.7 (1.9)
Daily Total Steps in Brisk Walking (min)	574.2 (670.1)	743.2 (1181.8)	574.9 (937.4)	470.1 (413.3)	440.3 (326.4)	450.7 (494.1)	435.7 (448)	337 (370.9)	333.4 (276.8)	198.8* **(202.4)**	20.1 (202.6)	267.6 (200.3)	102.9 (200.5)	68.8 (206.0)	82.8 (206.3)

B: Baseline; Btw-Cond. Diff: Between-Condition Difference; M: Month; MVPA: Moderate-to-Vigorous PA; PA: Physical Activity

Values Expressed as Mean (SE)

*: *p* < 0.05

As for the secondary outcomes, from baseline to Month 3, the [AVG Only] group had significantly less lean mass gain and less HDL cholesterol gain than [Waitlist Control] (net difference, 815.1 [992.2; 95% CI, 296.2-2442.3], gram; *p* = 0.04) and 7.2 [2.5; 95% CI, 4.4–11.3], mg/dL; *p* = 0.04), respectively.

In terms of the exploratory outcomes, from baseline to Month 3, both [Narrative + AVG] and [AVG only] groups had significantly more daily moderate PA (MPA) than [Waitlist Control], (2.8 [3.8; 95% CI, 1.5-9], minute/day; *p* = 0.04) and (5.6 [3.9; 95% CI, 1.2–12], minute/day, *p* = 0.01), respectively. For daily brisk-walking steps, [Narrative + AVG] exceeded [AVG Only] (net difference, 198.8 [202.4; 95% CI, 27.9-530.7], steps/day; *p* = 0.04).

All analyses were conducted with and without controlling for PAQ-C and Social Desirability as part of the sensitivity analysis. The Benjamini–Hochberg procedure was applied to account for multiple comparisons, and the results remained consistent.

### Intervention acceptance

Intervention acceptance was high in both [Narrative + AVG] and [AVG Only]. Despite challenges with downloading future AVGs, the game experience questionnaire showed consistently positive rating [Visit 2 = 3.45 (0.99), Visit 3 = 3.53 (1.11)]. The [Narrative + AVG] group reported high narrative immersion [Visit 2 = 3.10 (0.69), Visit 3 = 3.26 (0.85)], liking for the story [Visit 2 = 4.0 (1.05), Visit 3 = 3.89 (1.05)], and interest in watching additional episodes [Visit 2 = 3.64 (1.25), Visit 3 = 3.57 (1.40)]. A 96% correct response rate to narrative-specific questions confirmed most participants watched the animations. Research coordinators received consistently positive feedback during technical outreach and follow-up visits. These findings highlight strong intervention acceptance despite logistical challenges.

## Discussion

While the primary outcome, daily MVPA, did not show significant changes over time within each condition, the hypothesis received partial support from the post-hoc exploratory analysis. The [Narrative + AVG] group demonstrated more daily MVPA than the [Waitlist Control] group in the first half of the RCT.

Interestingly, the [AVG Only] condition experienced less lean mass gain and lower HDL cholesterol levels compared to the [Waitlist Control] in the first half of the intervention. Regarding exploratory outcomes, both [Narrative + AVG] and [AVG Only] produced higher moderate PA levels than the [Waitlist Control] during the first half of the RCT. Moreover, the [Narrative + AVG] group demonstrated more brisk walking steps than the [AVG Only] group during this period. The study was underpowered to conclusively determine differences between the [Narrative + AVG] and [AVG Only] groups. Nevertheless, participants’ acceptance of the intervention was high, indicating the need for a future fully powered study.

Although we did not observe a statistically significant effect for our pre-specified primary outcome, our study nonetheless offers a substantial contribution: integrating narratives with AVGs significantly increased MVPA and MPA among high-risk children, defined as being predominant racial and ethnic minorities with overweight or obesity, during the initial three-month period, compared to the [Waitlist Control] group. Specifically, the [Narrative + AVGs] group saw an additional 6.8 min of daily MVPA, with approximately 40% of this increase attributed to MPA and 60% to VPA. In contrast, the [Waitlist Control], while demonstrating a highly active status at the baseline, exhibited a declining trend in MVPA over the course of the RCT. Although these changes were most pronounced in the first half of the RCT and may not be clinically significant, they were nevertheless important, as even minor increments in daily MVPA can help establish and maintain long-term PA habits [63, 64].

While a 6.8-minute increase in daily MVPA and a 200-step increase in brisk walking may seem minimal, they can have a meaningful impact on the health of youth. Research shows that small, achievable increments in PA are often more manageable for children, potentially leading to more consistent and sustained PA behaviors over time [65]. Such increases can help children progressively build up to the PA guidelines. Even modest increases in daily PA can enhance cardiovascular fitness, reduce the risk of chronic diseases, and support healthy weight management in the long term.

The increase in PA coincided with the introduction of a video gaming console into the participants’ homes. This is noteworthy, considering these consoles could have been used for sedentary gaming, especially during the pandemic when PA levels declined globally, particularly among racial and ethnic minority families [66]. Family environments play a critical role in shaping children’s activity levels and overall health behaviors. If the introduction of a console led to a significant increase in sedentary activity, it could result in a substantial reduction in PA. Therefore, it is particularly significant that the introduction of the console in this study did not lead to a decline in PA levels, underscoring its potential as a tool for promoting PA rather than hindering it.

It is important to acknowledge that we did not account for growth rate and pubertal stage [e.g., Tanner stages [67]], which could impact the interpretation of the results. Future studies should collect such information as control. We did not observe a more direct relationship between MVPA and body composition. This may be due to the fact that we did not incorporate nutrients or dietary intake balanced across groups as confounding measures due to the small sample size [68–70]. Additionally, we did not focus on light PA, which could have a stronger fat mass-lowering effect than MVPA [71]. Furthermore, the relatively short RCT duration and the expanded age group may qualify these findings.

Unexpectedly, in the first half of the intervention, the [AVG Only] condition experienced less lean mass gain and lower HDL cholesterol levels compared to the [Waitlist Control]. This suggests that the activity promoted by AVGs alone may not have included sufficient intensity or resistance-based components, which are more effective for building lean mass and improving lipid profiles. These findings underscore that the type and quality of activity, rather than just its quantity, are critical in driving specific health outcomes.

The added benefit of incorporating child-friendly narratives into AVGs was inconclusive. While the addition of narratives led to significantly more MVPA compared to the waitlist control, and higher brisk walking steps compared to the non-narrative condition during the first half of the RCT, these effects were not sustained through the conclusion of the trial. A notable issue that may partially explain this finding is that despite providing instructional manuals at the first visit, more than half of the [Narrative + AVG] participants encountered difficulties downloading narratives through the Microsoft OneDrive app on their Xboxes. In our previous lab-based studies [19–23], we delivered the narratives directly to participants, ensuring seamless integration of storytelling and gaming elements. However, in this RCT, participants were required to retrieve the narratives themselves before resuming AVG play, creating barriers to effective narrative delivery and integration, possibly diminishing their long-term enthusiasm and engagement for PA in the later part of the RCT.

Moreover, downloading narratives required a high-speed Internet connection in participants’ homes, a significant issue given the pandemic’s exacerbation of the digital divide, particularly in underrepresented communities [72]. These factors likely contributed to the ambiguous results between the two conditions and the lack of sustained effects between baseline and Month 6, as many also encountered problems downloading the remainder of the five AVGs over the RCT. Comparatively, the AVG only condition offered a more straightforward user experience.

However, most, if not all, participants in the [Narrative + AVG] condition were able to access the narrative episodes despite various levels of delays. This is supported by evidence that participants were periodically queried to confirm they had indeed viewed the narrative animation three times during the intervention. These questions were specifically tied to the narrative element and could not be answered correctly unless the participants had watched the animation [e.g., What color is (A Supporting Character’s Name)’s hair? Purple / Orange / Green / Blue). Across the intervention, we observed a 96% correct response rate, suggesting that most participants in this group had indeed watched the narrative animation.

While we had originally planned for research assistants to visit participating families to deliver intervention materials when participants had trouble downloading the games and narratives, the pandemic shutdown prevented this. Future studies should investigate more effective (and less labor-intensive) methods for incorporating narratives into AVGs, such as autoplay of narratives before games start, ensuring an easy and engaging story interaction. To avoid lack of variety and boredom, delivering the AVGs in disk formats via mail could be more feasible and motivating for both AVG groups than downloading them.

### Strengths and limitations

Our study has multiple strengths. This is the first RCT to empirically test the 3–6 month effects of narrative impact on PA behavior, body composition, BMI%, and metabolic biomarkers among racial and ethnic minority children with overweight or obesity. We examined the impact of introducing AVGs and video game consoles into a free-living environment using objective and validated measurement devices and methods. Our RCT had a relatively long duration with multiple measurement timepoints, offering a nuanced understanding of the outcomes over six months. While we did not find statistically significant support for the primary outcome, unlike some similar studies [73], we detected multiple significant changes in PA from the post-hoc exploratory analysis. While our RCT was unexpectedly impacted by the pandemic, which also disproportionately impacted racial and ethnic minority youth [30], our findings are of particular benefit. Last but not least, we have accounted for multiple tests by applying the Benjamini–Hochberg procedure to minimize the likelihood of false-positive findings.

Our study also has several limitations. First, the pandemic significantly impacted participant recruitment and retention, lowering statistical power [32] and possibly contributing to the non-significant findings observed. While there was no group difference between the original recruits and those who completed the trial, as well as between the original and later expanded age groups, the RCT had a retention rate of around 60%. Future studies should consider enrolling more participants with creative retention methods [e.g., social marketing [74]] and extending the measurements to include pubertal status, dietary intake, and light PA. While a notable number of participants (*n* = 15 across three visits) provided fewer than three days of valid accelerometer data, the rate of providing complete PA data was not statistically significantly different across the three conditions (*p*s > 0.21). Additionally, the null effect observed in MVPA could be attributable to a ceiling effect at baseline, as participants already exhibited relatively high levels of PA compared to national prevalence estimates. Increasing intervention and follow-up duration to a year would better examine its effects on PA, body composition, BMI%, and metabolic biomarkers and provide a more comprehensive picture of the AVG and narrative impact.

In terms of the gameplay, the narrative and AVG delivery mechanisms need refinement to ensure better integration with existing AVGs and easier gaming access, especially given the disparities in Internet connection speed affecting story delivery. Our attempt to objectively assess active and sedentary gameplay using an automated script to track game console usage (and therefore track participant engagement with the AVGs) was hindered by multiple AVG developers’ modifications to their code in the Xbox game library during the pandemic. This resulted in the inability to retrieve objective playtime data, with no timeline provided for resolving the issue. Consequently, effective documentation and retrieval of gameplay and usage data information were not possible. As a result, we were unable to compare active versus sedentary gameplay or confirm that the observed increase in MVPA and overall exercise was directly attributable to AVG play due to the lack of real-time documentation of gameplay intensity and engagement.

The lack of detailed evaluation of growth and pubertal data limited our interpretation of changes in body composition [75, 76]. Some of the counterintuitive findings, such as the higher lean mass gain observed in the [Waitlist Control] group, highlight the need for more nuanced and rigorously designed, higher-powered research to better understand these unexpected outcomes.

Despite this, our sensitivity analysis and the Benjamini–Hochberg procedure revealed consistent results, reinforcing our confidence in the robustness of the results.

Lastly, the cost of the Xbox and Kinect bundle may be prohibitively expensive for underrepresented families of lower social economic statuses, especially following the price hike after Microsoft’s decision to discontinue Kinect products. Future studies might consider switching to a more accessible AVG delivery system, such as apps and virtual reality headsets.

## Conclusions

Both AVGs paired with narratives and AVGs alone failed to consistently improve daily MVPA or secondary outcomes across the 6-month RCT. However, exploratory analysis suggested that the integration of narratives positively influenced multiple PA outcomes compared to both the waitlist control and AVG-only groups. However, these findings should be interpreted with caution given the multiple variables and comparisons involved in the study. The long-term evidence for the added advantage of narratives was inconclusive, likely due to implementation challenges and insufficient statistical power among underrepresented populations, which may have contributed to the less-than-expected effects. These findings provide valuable insights and emphasize the need to address these challenges in future research to support a fully powered study.

## Electronic supplementary material

Below is the link to the electronic supplementary material.

Supplementary Material 1

Supplementary Material 2

Supplementary Material 3

Supplementary Material 4

## Data Availability

The datasets used and/or analyzed during the current study are available from the corresponding author on reasonable request.

## Abbreviations

AVG: Active Video Games
MPA: Moderate Physical Activity
MVPA: Moderate to Vigorous Physical Activity
PA: Physical Activity
RCT: Randomized Clinical Trial
VPA: Vigorous Physical Activity
