# Supplementary material for: The impact of narratives and active video games among black and hispanic children with overweight and obesity: a randomized controlled trial
Source: Int J Behav Nutr Phys Act. 2025 May 26;22:60. doi: 10.1186/s12966-025-01756-1 (PMC12107869; doi:10.1186/s12966-025-01756-1)
Supplement: Supplementary file 1 — Supplementary Material 1 [file 12966_2025_1756_MOESM1_ESM.docx]

Supplementary Table 1: Between-Condition Differences in Physical Activity Levels, Body Composition, BMI percentile, and metabolic biomarkers through Linear Mixed Model (Second Half of RCT—Month 3 to Month 6 Comparisons)

|  | Group A: Narrative + AVG | | |  | Group B: AVG Only | | |  | Group C: Wait List Control | | |  | Btw-Cond. Diff:  A vs. B  3 M-6 M Change | Btw-Cond. Diff:  A vs. C  3 M-6 M Change | Btw-Cond. Diff:  B vs. C  3 M-6 M Change | |  |  |
| --- | --- | --- | --- | --- | --- | --- | --- | --- | --- | --- | --- | --- | --- | --- | --- | --- | --- | --- |
| Study Outcome | | B | 3 M | 6 M |  | B | 3 M | 6 M |  | B | 3 M | 6 M |  |  |  |  |  | |
| **Primary Outcome** | | | | | | | | | | | | | | | | |  |  |
| Daily MVPA (min) | | 41.8  (17.3) | 44.9  (20.9) | 41.8  (14.9) |  | 39.6  (14.9) | 41.1  (18.3) | 41.3  (22.8) |  | 47.3  (22) | 43.5  (17.8) | 42.3  (18.2) |  | **-3.3***  **(4.5)** | -1.8  (4.4) | 1.4  (4.6) | |  |
| **Secondary Outcomes** | | | | | | | | | | | | | | | | |  |  |
| Total Fat (g) | | 26269.9  (11513.8) | 26816.5  (11584.1) | 27094.5 (11032.1) |  | 27101.5 (10689) | 26745.1 (11173.7) | 26746.6 (11855.4) |  | 25503.5 (8479.7) | 26338.2 (8968.2) | 27786.4 (9755.6) |  | 276.6 (673.4) | **-1170.1* (666.5)** | **-1446.6* (685.5)** | |  |
| Total Lean (g) | | 35416  (8876.3) | 37055.5  (9310.5) | 37922.7 (10079.9) |  | 34269.6 (6809.4) | 35463.2 (7744.8) | 36300.7 (8028.3) |  | 33848.6 (7418.5) | 35857.3 (9376.8) | 36813.2 (10348.6) |  | 29.7 (545.4) | -88.7 (539.8) | -118.4 (555.2) | |  |
| Total Region Fat (%) | | 40  (8.5) | 39.6  (8.9) | 39.7  (8.8) |  | 41.5  (7.5) | 40.5  (7.9) | 39.8  (9) |  | 40.9  (6.2) | 40.5  (6.2) | 41.2  (6.2) |  | 0.8  (0.6) | -0.5  (0.6) | **-1.3***  **(0.6)** | |  |
| BMI % | | 96.4  (2.7) | 95.9  (3.7) | 96.1  (3) |  | 96.2  (3.9) | 96.3  (3.2) | 95.8  (3.9) |  | 96.5  (4) | 96.9  (3.1) | 96  (4.9) |  | 0.7  (0.4) | 1.2  (0.7) | 0.4  (0.7) | |  |
| Fasting Insulin | | 16.6  (11.5) | 16.9  (10) | 16.8  (16.4) |  | 13.1  (7.1) | 21  (17.2) | 14.3  (8.5) |  | 18.6  (12) | 23.8  (27.6) | 20.9  (22.5) |  | 6.5  (4.4) | 2.8  (4.3) | -3.7  (4.4) | |  |
| Glucose | | 91.3  (9.3) | 89.9  (8.8) | 88.8  (11.1) |  | 89.5  (8.7) | 91.9  (6.8) | 89.7  (8.1) |  | 93.8  (23.3) | 96.8  (32) | 96.7  (42.9) |  | 1.2  (3) | -0.9  (3.9) | -2.1  (3.9) | |  |
| Cholesterol | | 164.3  (23.7) | 159.5  (29.9) | 158.2  (21.1) |  | 163.1 (33.2) | 153.7  (28.4) | 160  (39.4) |  | 163.2  (32.3) | 164.7 (35.4) | 159.6  (36.5) |  | -7.5  (7.1) | 3.8  (7.4) | 11.4  (8.7) | |  |
| HDL cholesterol | | 51.2  (10.4) | 52.1  (14.4) | 52.9  (10.3) |  | 49.3  (13.7) | 47.3  (9.8) | 48  (9.9) |  | 48.8  (10.3) | 54  (12.2) | 51.8  (13.6) |  | 0.1  (2.8) | 3  (3) | 2.9  (2.8) | |  |
| LDL cholesterol | | 95.7  (22.8) | 89  (26.2) | 89.1  (20.5) |  | 96.7  (25.1) | 89.7  (26.1) | 94.3  (32.2) |  | 96  (26.2) | 92.2  (27.1) | 89.2  (27.7) |  | -4.5  (6) | 3.1  (5.7) | 7.6  (7.2) | |  |
| Triglycerides | | 84.9  (32.2) | 97.2  (64.4) | 77.3  (34.2) |  | 80.1  (23.7) | 79.8  (23.5) | 86.1  (28.3) |  | 92  (34.2) | 92.5  (44.9) | 93.1  (37.3) |  | **-26.2***  **(12.5)** | -20.5  (13.4) | 5.6  (10.5) | |  |
| C-reactive protein | | 3.2  (4.2) | 3.7  (3.5) | 4  (4.7) |  | 3.3  (2.4) | 3.6  (3) | 2.6  (2.2) |  | 2.8  (2.9) | 2.7  (2) | 3  (3.6) |  | 1.4  (0.8) | 0.1  (0.8) | -1.3  (0.7) | |  |
| **Exploratory Outcomes** | | | | | | | | | | | | | | | | |  |  |
| Daily Vigorous PA (min) | | 10.1  (5) | 12.7  (10.7) | 11.3  (7.8) |  | 10.7  (5.8) | 9  (5) | 9.9  (7.4) |  | 12.9  (9.2) | 11.5  (6.8) | 10.7  (6.8) |  | -2.3  (2.1) | -0.5  (2.1) | 1.8  (2.1) | |  |
| Daily Moderate PA (min) | | 31.7  (13.3) | 32.2  (15.3) | 30.5  (11.5) |  | 28.9  (10.5) | 32.1  (13.7) | 31.4  (15.7) |  | 34.3  (13.9) | 32  (12.8) | 31.6  (13.2) |  | -1.0  (3.1) | -1.3  (3.1) | -0.3  (3.2) | |  |
| Daily Brisk Walking (min) | | 5.3  (6.2) | 6.9  (10.8) | 5.3  (8.5) |  | 4.4  (3.8) | 4.1  (3) | 4.2  (4.6) |  | 4.1  (4.2) | 3.1  (3.4) | 3.1  (2.6) |  | -1.7  (1.8) | -1.6  (1.8) | 0.1  (1.8) | |  |
| Daily Total Steps in Brisk Walking (min) | | 574.2  (670.1) | 743.2  (1181.8) | 574.9  (937.4) |  | 470.1  (413.3) | 440.3  (326.4) | 450.7  (494.1) |  | 435.7  (448) | 337  (370.9) | 333.4  (276.8) |  | -178.6 (193.0) | -164.6 (191.1) | 14.0  (196.5) | |  |

B: Baseline; Btw-Cond. Diff: Between-Condition Difference; M: Month; MVPA: Moderate-to-Vigorous PA; PA: Physical Activity

Values Expressed as Mean (SE)

*: *p* < 0.05
