# Supplementary material for: The impact of narratives and active video games among black and hispanic children with overweight and obesity: a randomized controlled trial
Source: Int J Behav Nutr Phys Act. 2025 May 26;22:60. doi: 10.1186/s12966-025-01756-1 (PMC12107869; doi:10.1186/s12966-025-01756-1)
Supplement: Supplementary file 2 — Supplementary Material 2 [file 12966_2025_1756_MOESM2_ESM.docx]

**Supplementary Analysis of the Comparison of Group Means between Month 3 and 6**

*Results*

In terms of the primary outcome variable, daily moderate-to-vigorous PA (MVPA), our post-hoc exploratory analysis indicated that from Month 3 to Month 6, the [AVG Only] group had more daily MVPA than [Narrative+AVG] (net difference, 3.3 [4.5; 95% CI, 1.7-10.7], minute/day, *p*=0.03).

As for the secondary outcomes, from Month 3 to Month 6, the [Narrative+AVG] group gained less triglycerides, -26.2 [12.5; 95% CI, -46.7 - -12.2], mg/dL; *p*=0.04) than [AVG only]. Additionally, [Narrative+AVG] gained significantly less fat mass than [Waitlist Control] (net difference, -1446.6 [685.5; 95% CI, -2790.2 - -103.0], gram; *p*<0.01. On the other hand, [AVG only] gained significantly less fat mass and had a lower Total Region % Fat compared to [Waitlist Control] (net difference, -1170.1 [665.5; 95% CI, -1916.6 - -77], gram; *p*<0.01 and -1.3% [0.6; 95% CI, -2- -0.3]. %; *p*=0.02, respectively). Please refer to Supplementary Table 1 for details.

*Discussion*

During the second half of the RCT, the [AVG Only] group produced more daily MVPA. Both [Narrative+AVG] and [AVG Only] resulted in significant fat mass reduction compared to the [Waitlist Control], with the [AVG Only] group also exhibiting lower Total Region % Fat than the [Waitlist Control]. Additionally, during this time, the [Narrative+AVG] condition significantly reduced triglyceride levels compared to the [AVG Only] group.

Additionally, regardless of the narrative addition, introducing AVGs significantly curtailed fat mass gain in both the [Narrative+AVG] and [AVG only] groups, compared with [Waitlist Control] during the second half of the RCT. Notably, the [AVG only] group also exhibited a 1.3% reduction in total regional fat percentage relative to the [Waitlist Control] Group. While these reductions may not be clinically significant, it is a promising trend. This stage of life marks a critical period of growth and development, typically accompanied by a continuous increase in average percent body fat (1) and a sharp decline in PA (2). Effectively managing fat mass gain during this phase is vital for preventing long-term chronic conditions, enhancing psychological well-being, reducing insulin resistance risk, and improving fitness, endurance, and immune function (3). Additionally, this result is particularly significant in the context of the pandemic-related weight gain among children globally (4).

The [Narrative+AVG] condition significantly reduced triglyceride levels compared to the [AVG Only] group in the second half of the RCT. This reduction suggests that the narrative may have encouraged certain types of exercise that might have led to improved lipid metabolism and the observed triglyceride reduction. However, as noted in the main text, we encountered multiple challenges during the execution of the RCT. Although we believe that the narrative could have contributed to some of the observed effects, further testing is required.

.

References

1. Guo SS, Chumlea WC, Roche AF, Siervogel RM. Age- and maturity-related changes in body composition during adolescence into adulthood: the Fels Longitudinal Study. Int J Obes Relat Metab Disord. 1997;21(12):1167-75.

2. Troiano RP, Berrigan D, Dodd KW, Masse LC, Tilert T, McDowell M. Physical activity in the United States measured by accelerometer. Medicine & Science in Sports & Exercise. 2008;40(1):181-8.

3. Whitlock EP, O'Connor EA, Williams SB, Beil TL, Lutz KW. Effectiveness of weight management interventions in children: a targeted systematic review for the USPSTF. Pediatrics. 2010;125(2):e396-418.

4. Chang TH, Chen YC, Chen WY, Chen CY, Hsu WY, Chou Y, et al. Weight Gain Associated with COVID-19 Lockdown in Children and Adolescents: A Systematic Review and Meta-Analysis. Nutrients. 2021;13(10).
